# Monthly Periscope

**Published:** 1853-11

**Authors:** 


*Monthly Periscope.—Notes in the Dissection Room.*—A brain which had been used in demonstrating, fell suddenly apart while being carelessly handled. This brain was that of an idiot person, and had been preserved since some time last summer, in alcohol. At the time of the *autopsia cadaveris*, no unusual appearances were presented, save the smallness and unusual number of the convolutions. All the external appearances, both superficial, and on the base of the brain, were entirely normal. In the course of the demonstration, only the left hemisphere had been pared down to the ventricular cavity. This was entirely normal. After the accident which, (tearing across the corpus callosum and floor of the third ventricle,) rendered it useless for anatomical purposes, I found the following abnormal changes in the right hemisphere, which sufficiently accounted for idiocy: The cavity of the left lateral ventricle was scarcely distinguishable. The corpus striatum and optic thalamus were *shredded* by some process like interstitial absorption, leaving these bodies a mass of disintegrated fibres. Outside the ventricle, was a cavity extending the whole length of the hemisphere, at a distance from its lateral surface varying from a half inch to an inch; this cavity was deep, and separated the commissural from the peripheral portion of the brain, almost entirely. The texture of the white matter of the brain, (medullary portion,) was reticulated and honeycombed, the little cavities being filled with a clear gelatinous fluid.

It will be seen that we could not arrive so accurately at the pathology of this interesting case, as we might have done had the brain been fresh, but it has, nevertheless, considerable physiological interest. The subject was not paralytic, and the muscular system was, throughout, well developed. How an amount of disease which destroyed the integrity of the whole left hemisphere of the brain except its base, could exist without paralysis, seems inexplicable, though other cases not dissimilar have occurred. The New Hampshire case of the iron bar through the brain, and Dr. Detmold's case of abscess of one of the hemispheres, when he carried the scalpel deep into the substance of the brain to give exit to purulent matter, are alike parallel wonders.

*Unusual distribution of arteries.*—In a subject of otherwise usual conformation, we found the innominata wanting. The right carotid passed upward by a distinct trunk. Next to it on the left arose the left carotid, and left subclavian arteries, by their usual distinct trunks, and following their usual and normal distribution. The right subclavian left the arch of the aorta at its downward curvature, starting from it to the left of the left subclavian, passing upward and inward, attaining the median line directly behind the trachea, at a point opposite the upper border of the thyroid body, then dipping downward and outward, it reached in the right axilla its usual course.

Any operation for tying this right subclavian at the usual place would have been a failure. The right lung of this subject had but two lobes.

*Reid's method of reducing Dislocations of the Hip-joint.*—The following dissection and experiment with reference to Dr. Reid's method, will be interesting to those who have watched the controversy in regard to it:

The glutæus maximus and medius having been removed, and the obturator muscles being exposed, a slit was made in the capsular ligament between the obturator internus, and gemellus superior, and the head of the femur dislocated upward and backward through the slit in the capsule. The effect of the dislocation was to put upon the stretch the obturators, the two gemelli, the pyriformis, and the quadratus femoris. On making extension in a right line downward, the opening between the muscles closed tightly around the head, offering a very firm resistance to reduction; and as traction on the limb was increased, the muscles grasped the bone more firmly. It was evident that in the living subject, the resistance of the muscles increasing with the extension, would interfere with, and forbid reduction until they should become tired out, or torn across by the force applied. But on flexing the limb upon the body, these muscles became flaccid, and a slight rotatory motion of the foot, while the thigh was still flexed on the body, restored the bone to its place with a readiness which excited the admiration of all who witnessed it. The experiment was repeated on another limb with equal success.

*Benique's mode of treating Stricture.*—The Louisville "Western Journal" has a letter to Prof. Gross, from Elkanah Williams, M. D., of Cincinnati. Our readers will recollect seeing in this Journal a review of Prof. Paul F. Eve's monograph on Benique's method, with some extracts. The principle involved is that of *rapid* dilatation. In a case witnessed by Dr. Williams, in the practice of M. Nelaton, a stricture of twenty-one years' standing, was cured in twelve days. The stricture was traumatic. Benique's method consists of thirty-six solid metallic bougies, graduated from one and one-third to five lines. As many of these are introduced at one sitting as possible, rapidly increasing the size, and commencing on the succeeding day with the size last used. In M. Nelaton's case, this management was eminently successful, and on the thirteenth day after commencing the treatment, a medium sized catheter was passed with ease. This new mode of treating stricture recommends itself to our surgeons, and promises much in the cure of a very intractable affection, manifesting much disposition to return after an apparent cure.

*New method in Fractures.*—From the same source we learn of a new and ingenious substitute for the paste bandage in dressing fractured bones. The bandage of Scultetus, (or many tailed bandage,) is slightly wetted with water, and then each slip is sprinkled with perfectly dry and finely pulverized plaster of Paris, and instantly applied. Thus we have an immovable plaster cast about the limb, which has the merit of great convenience, as it dries and becomes firm instantly, thus avoiding the chance for displacement which exists during the application and drying of the ordinary paste bandage. A roller applied to the limb before the bandage is laid on, prevents the irritation of the skin which would otherwise ensue. The entire want of ventilation is the apparent objection to this bandage, but it is one rather apparent than real, as we judge from some knowledge of the paste bandage. It uniformly happens that the atrophy or shrinking of the limb under the dressing, is sufficient to afford all necessary access of air, and facility for evaporation of the secretions of the skin.

*Births, Marriages, and Deaths.*—During the months of July, August, and September, there were, in the city of New York, 5077 births, while the number of deaths was 7111, thus showing an excess of deaths to the amount of 2034.

It might at first glance appear that this was a casual circumstance—a mere coincidence affecting these three months—that owing to sickness peculiar to the season, or to the temporary residence of a great number of strangers, the excess was only a transient phenomenon, to disappear on the return of a healthy season. But this is not altogether the case. Undoubtedly among the many thousands who have, during this season, visited New York on temporary errands of pleasure or business, some have perished to swell the mortuary list. But the city has been healthy. No epidemic has prevailed, and the amount of summer and autumnal disease has been less than usual. We are to look, then, elsewhere than to influx of strangers, or unusual disease, to find the cause of this startling result. That it is startling is evident from the brief but pregnant calculation, that in the period of sixty-five years this excess of deaths over births, would sweep from the earth the whole vast population of the fourth city in the world, were it not for the constant accession of immigrants from Europe and the rural districts.

Some one has said, that "great cities are great sores upon the body-politic." The tendencies of city life are toward a decrease of population — not only in New York, but in all other great cities, does this hold true, and in all of them we find an excess of deaths over births. The deficit thus created is filled, in the case of New York, from two sources — immigration from Europe, and from the rural districts. In the great cities of Europe, the former cause does not operate; and it is the surrounding districts which maintain their population, by sending in a yearly holocaust, to fill the gap of those who have perished.

In spite of this exhausting waste, the population of New York has increased one hundred and fifty thousand in the last ten years. According to the average of the past three months, the excess of deaths over births must have been some fifty thousand, so that the actual accession to the population of New York, in the last ten years by immigration from Europe, from the rural districts, and from births, has been nearly or quite two hundred thousand souls.

Who can estimate the importance of a thorough system of registration of births, marriages, and deaths, as developed by these results? The present law gives to the city of New York the benefits of such registration, and the facts which we have thus briefly enumerated, illustrate the additions to our knowledge to be gained by such a law, so far as the statistics of three months have any value. But when these shall have been verified and corrected by the statistics of succeeding years, when we shall see rising from those figures some tangible results in the shape of sanitary laws, and of development of great principles in political and moral economy, we shall be able, in some measure, to appreciate the benefits to be derived from a comprehensive system of registration.

There is in this direction a field for research spreading vast and far before us—its limits hid in that which is unknown, but destined to reward the reaper of its harvest with many sheaves of good, of benefits to the race, and of honor to those who labor in its gathering. To the medical profession it promises most, and its members should be foremost in advancing their interests. The credit of the profession requires that its influence should be felt, in guiding and framing laws of this character. We have not as yet any sufficient law of registration. The one proposed and urged through the legislature, a few winters since, by the venerable Dr. Backus, of Rochester, then a senator, was lacking in some minor details, which interfered with, and obstructed its operation. Only some amendments to the law framed by Dr. Backus are needed. These amendments are those relating to details only, and are suggested by some practical knowledge of the workings of the law.

When it was first passed, we, (then practicing in the country,) succeeded, by personal solicitation and aid, in securing from the School District Clerks, in our own town, a full report. But never again. The clerks urged, very plausibly, that the law made an onerous addition to their labors, for which they received insufficient pay. Aside from the labor of preparing the report, (no small task in a large district, and especially vexatious in what are called "joint-districts,") they were obliged to give it in to the town clerk. They had no other official intercourse with that officer. Frequently it required a special journey of half a dozen miles to transmit it, and not infrequently these minor vexations entirely prevented any return whatever. Had they been directed simply to embody a full report of births, marriages, and deaths, in the annual report of the trustees to town superintendents of schools, it would have been done much more easily. To this functionary every district in the town reports almost unfailingly. Upon their report, and its completeness and accuracy, depends the school money for the ensuing year. If the law were to make this registration a necessary part of the annual reports of trustees to town superintendents, the pecuniary interest of the district would require the performance of the duty, and would insure a full and accurate body of statistics.

Convenience, too, is in favor of this change. The town clerks have little intercourse with the county clerks, to whom they were obliged to report in turn, and very generally they were a class of men, unappreciative of the benefits of the law, and incapable of collating properly the district returns. Hence it happened, that the statistics were often lost on the way, and never reached the county clerk. The change which we have proposed, would transfer the business of making the district reports from the clerks to the trustees—it would enable these latter to embody their statistics in returns of a somewhat similar character, which are annually made—it would make it essential to the pecuniary interest of the district, that there should be a full report; and, finally, it would transfer the function of collating and condensing the district reports, to the town superintendent, who is almost universally a man of some education and sympathy with such movements.

We have no especial fondness for this plan because it is our own, but we have an anxiety to see *some* provision made to secure the benefits intended to be conferred by the present law. The want of it is an offense to our patriotism, an insult to the intelligence of the state. We hope that the incoming legislature may find time to look to this matter a little. When they have given us such a law, and with it one legalizing anatomical pursuits, we shall be content to endure the absence of all legal support and recognition as a profession, which they have inflicted upon us. The few statutory enactments for which the medical profession pleads, are rather for the general good than for its own.

*Spiritual Manifestations.*—When a new theory is announced, or a new discovery claimed, we may, not unfrequently, arrive at its truth by estimating its value. All extension of knowledge must result in good. If only evil results are attainable from a new theory, then we may conclude that it is in itself false and unworthy.

Acting on this belief we have not ourselves been at the pains to experiment in this matter of spirit-rappings, and our own experience in them is confined to sundry divings under tables, and bruising of shins over chairs in the dark one night, in search of the cause of certain loud raps which woke us from our slumbers. These noises, though inexplicable at the time, were subsequently explained by reference to very natural causes.

It is claimed on the part of the advocates of this new doctrine, that a new channel of communication has been opened between the world unseen, and mortals. As the result of this intercourse, we are now favored by literary communications from the spirits of departed great men, while friendly messages are dispatched to and from them, with the greatest facility. Some of these productions we have perused. St. Paul, in particular, is sending down codicils to his Epistle to the Romans in very frequent installments. It is worthy of remark that this saint seems to have lost that nervous and vigorous literary style which characterized his earlier productions, and now utters only the most absurd of platitudes. He exhorts some afflicted Mary Ann to "be patient;" tells her that the "great Harmonia" will soon commence, and that if she will only hold her temper and slip quietly out of the world at the first opportunity, all will be pleasant on the other side of Jordan. This falling off in literary merit is not confined to St. Paul. Most of the inspired writers, as well as the spirits of more recent great men, seem to have retrograded most shockingly since their departure. They are anything but "just men made perfect."

Another peculiarity of these communications is the mode of their manifestation. The most dignified of spirits do not hesitate to perform the most boyish tricks. Franklin tips over tables, Washington breaks crockery, and Lord Bacon spins chairs around on a single leg. We do not set any very high estimate on our own personal dignity, but we should really be ashamed to be found engaged in such very trivial occupations, and if our exodus from this planet is to be the beginning of similar avocations on our part, we shall pray for an indefinite postponement of our departure, and a long continuance here in the practice of medicine.

We have always understood that there was no aristocracy beyond the grave; that the only distinction there was one of merit, and that liberty, equality, and fraternity, were the chiefest glories of the world to come. That such is the case is most evident, from the fact that the most dignified of defunct philosophers, statesmen, and saints, are always ready to hold a conference with any bread-and-butter school girl who may summon them to consultation in her love matters. Our mediums are never at a loss for advisers. They resort at once to the spirit-world, and consult its denizens with the utmost freedom as to who stole their umbrella.

Such, we believe, are the only tangible results of all the spirit-rapping and table-turning. But in spite of the ridiculousness of these manifestations, very many minds, otherwise strong and healthy, have been deluded by them, and not only the ignorant and superstitious, but the learned and acute are found among their believers. Indeed, it is said that one of the most eminent jurists in this state is about to publish a work on the subject, and is to give therein the communications he has received from departed spirits. Of course it is not to be expected that a book from Judge Edmonds will be of that empty and worthless character which has marked the productions of lesser mediums. It will, of course, partake of that strength and merit which belongs to a mind naturally clear, and still sane on every other subject. But should this forthcoming work possess the very highest order of talent, it will only prove that the character of the communications received, depends on the mind through which they pass, and not on the source whence they ostensibly proceed. We think that a careful examination of the phenomena presented, will present an entire want of that depth and originality which should characterize communications from so important a source; while the methods by which the alledged spirits choose to evince their presence and power, are undignified and unworthy of credence. The phenomena of raps has already been explained by the developments of our colleague in his examinations of Mrs. Fish and Fox, the great originators of these raps.

In that case, it was evident that the raps were due to natural causes, and originated in deception and fraud on the part of those mediums. It has since been urged, that although these women may have been imposters, it is difficult to suppose that others who produced the same sounds with no ends to serve thereby, and who were persons of respectability and veracity, should have been engaged in like deception. We are aware that persons of this description have in apparent good faith acted as mediums in various ways, some by rapping, some by table-turning, and some by writing, or other modes of manifestation.

So far as our own knowledge of these persons goes, they are all females, and we attribute all these phenomena occurring in such cases, to that moral obliquity which belongs to hysteria. If this position be true, it brings this subject within the immediate field of medical research, and opens an interesting page for inquiry. We believe that there is in all cases of hysteria some degree of deception on the part of the patient. Probably in very many cases the patient is scarcely aware of such deception, but we nevertheless believe that a hysterical convulsion, or coma, is incompatible with a strict regard for truth. All women of strong mind and strict moral sense, can control their nervous systems to such an extent as to prevent these abnormal actions. This is our own theory of hysteria. It is one which has many apparent objections, which we do not choose to stop to argue now, inasmuch as it is not necessary to our present purpose.

Many of the phenomena of spiritualism are certainly hysterical. A girl in an excited and unnatural condition of mind, takes her pen in her hand to be moved by spirits in reply to questions to be asked. She soon becomes nervous, and growing excited, turns pale, and begins to write. Perhaps at the first trial the pen makes only some irregular attempts at writing, but the natural appetite for the marvelous, the indirect compliment to her vanity, and her increasing nervousness, combined with that strange yielding to erratic impulses, (involving more or less departure from truth,) which characterizes hysteria, all lead her on to a more perfect success at the second trial. Once engaged in it, and known as a spirit-rapper, her own reputation for truthfulness requires that the imposition should be maintained. We say imposition but it is probable that the medium herself is as often imposed upon, and deceived by her own unmanageable and ill-governed nervous system, as she is herself the willful imposter. Any one who has witnessed the usual willfulness and perversity of hysteria, can understand this reasoning. It is of the same chapter of phenomena as that involved in "having the power," in the gift of tongues, and the faculty of miraculous cures, so common among religious fanatics and rogues.

Raps are given under the same circumstances. They are involuntary in the same sense as is that rapid, vibratory motion of the heel, produced when the toes rest lightly on the floor, the body being in a sitting position. In this very common motion, the nervous action is reflex, and not volitional, but is nevertheless very readily stopped when the will is brought to bear upon it. A worthy physician in Massachusetts, finding that he, too, could write without volition by calling on the spirits, obtained answers so ridiculous and contradictory, that he looked about for some natural cause, which he fancied he discovered in what he called "detached vitalized electricity," which we suppose means irregular nervous action, *alias*, hysteria. We can only reason *a priori*, but we should like to witness the effect of tr. valerian, tr. assafœtida, $\overline{aa}$ ℥j., on a case of honest spirit-rapping or writing.

*Cholera.*—It is announced that the cholera is again upon its way from the east to revisit our country. The exemption from disease which has characterized the past summer, can hardly be predicted of the coming year. A year ago the public mind had scarcely recovered its cheerfulness after one of the most fatal of these visitations. The area of cholera for the year 1852, was very limited. The cities of Buffalo and Rochester suffered beyond any other of our large towns, and it would scarcely be considered exaggeration to say, that the epidemic was confined to these two places. Upon them the scourge fell heavily; and nowhere would a deeper dismay be felt at its return. Other cities as much exposed, situated on the great lines of travel, and with no better sanitary precautions, escaped entirely. In New York, only isolated cases occurred from time to time, and at no period did it prevail as an epidemic. The other towns situated on the Central Railroad, and those lying to the west on the great lakes, escaped. Sandusky, which the year before had suffered beyond example, was healthy. A mysterious Providence seemed to direct its judgments on Rochester and Buffalo alone. We have, however, always been convinced, that however occult the proximate causes of cholera, its predisposing causes were more within the reach of knowledge. The instances wherein cholera invaded the dwellings of the rich, and of those who were not exposed to the action of the ordinary causes, such as filth and vegetable decomposition, are evidently only exceptions to the general rule. Wherever impure air, personal filth, and insufficient or improper food were combined, was the peculiar habitat of cholera.

It is, then, to sanitary measures and precautions that we must look for safety. It is possible that the progress of the pestilence may be stayed upon the other shore of the Atlantic, but our city authorities should not rest upon any such possibilities. Every effort should be made to promote cleanliness in the streets, and on private premises. The drainage of low places should not be deferred until the epidemic is upon us; and should this disease not reach us, the outlay of money and time will be amply remunerated by the additional comfort and immunity from ordinary disease which will be attained.

The returns for the city of London, issued by the Registrar General, indicate that true Asiatic cholera is now present in that city; and it is evident, from the tone of the medical periodicals, and from the care and pains bestowed upon the subject by the authorities, that a painful attention is already awakened to the probability of a severe epidemic. Once before, the cholera arrived at London in the fall, occasional cases only occurring during the winter, until on the opening of warm weather in the spring, it was developed in the greatest malignity.

As on that occasion, the cholera now haunts the purlieus of the docks, and the lower and poorer portions of the city. During the week ending September 24th, there were 29 deaths from cholera, and 89 from diarrhœa. Of the 29 cases, 16 are specified as Asiatic cholera. The whole number of deaths from all causes was 969, which is less than the average by nearly three hundred. Of these deaths nearly one eighth were from diarrhœa and cholera. The number itself is trivial, but the time of its occurrence, the character of the cases, with the mode of its importation from the north of Europe, where it is now prevailing in a malignant epidemic form, give to the number itself, though small, a deep significance. The lessons of previous epidemics teach us this fact, and warn us of the coming danger.

*Prof. Draper's Introductory.*—This production appears in a full report in the New York Daily Times. It is a very able defense of anatomical pursuits, and we are rejoiced to see the awaking interest in the profession on this point. Prof. Draper shows the injustice and folly of the present law, and demands an enactment which will legalize anatomical dissections. There are two good things in Prof. Draper's Introductory: One is that it says nothing about inductive reasoning, Lord Bacon, and all that. The other good thing lies in the fact, that he has attacked an error which is tangible and within reach, and which his introductory will help to abolish. As a student we were a sturdy violator of the present law; as a physician we suffered from the gradual departure of the anatomical knowledge we had acquired at no little risk; and as a demonstrator we have been made to feel most sensibly the necessity of some change for the better in this matter. We are, therefore, willing to follow the lead of Prof. Draper, or any other sensible man, in any practical effort to substitute a better law for the present. S. B. H.

